# Covid 19: Diet Composition and Health

**DOI:** 10.3390/nu13092980

**Published:** 2021-08-27

**Authors:** Dorota Skrajnowska, Maja Brumer, Sylwia Kankowska, Magdalena Matysek, Natalia Miazio, Barbara Bobrowska-Korczak

**Affiliations:** Department of Bromatology, Medical University of Warsaw, Banacha 1, 02-097 Warsaw, Poland; dorota.skrajnowska@wum.edu.pl (D.S.); s072231@student.wum.edu.pl (M.B.); s073006@student.wum.edu.pl (S.K.); s077164@student.wum.edu.pl (M.M.); s077158@student.wum.edu.pl (N.M.)

**Keywords:** virus SARS-CoV-2, nutritional status, dietary components, defense mechanism

## Abstract

The virus severe acute respiratory syndrome coronavirus 2 (SARS-CoV-2) causes the disease coronavirus disease 2019 (COVID-19). The cumulative number of cases reported globally is now nearly 197 million and the number of cumulative deaths is 4.2 million (26 July to 1 August 2021). Currently we are focusing primarily on keeping a safe distance from others, washing our hands, and wearing masks, and the question of the effects of diet and diet-dependent risk factors remains outside the center of attention. Nevertheless, numerous studies indicate that diet can play an important role in the course of COVID-19. In this paper, based on select scientific reports, we discuss the structure and replication cycle of SARS-CoV-2, risk factors, dietary standards for sick patients, and the roles of the microbiome and dietary components supporting the immune system in preventing COVID-19.

## 1. Description of the SARS-CoV-2 Virus

**Figure 1:**
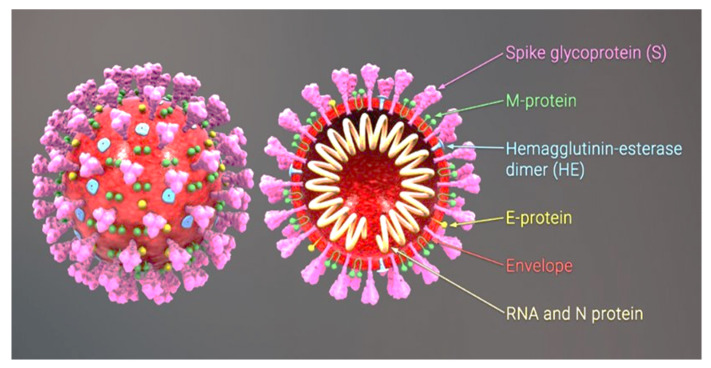
The structure of a coronavirus [4]. E—envelope; M—membrane; S—spike glycoproteins, which allow viral particles to bind to receptors on the host cell membranes.

The virus SARS-CoV-2 (Severe Acute Respiratory Syndrome Coronavirus 2) causes the disease COVID-19 (Coronavirus Disease 2019). The cumulative number of cases reported globally is now nearly 197 million and the number of cumulative deaths is 4.2 million (26 July to 1 August 2021) [1]. The original carriers of SARS-CoV were bats. In 2019 the interspecific barrier was breached, with tragic consequences. The transmission of the virus from bats to humans most likely involved an intermediate host, i.e., the Asian palm civet, sold at wildlife markets in the Chinese province of Wuhan [2]. The SARS-CoV-2 virus is transmitted by airborne droplets and as an aerosol. Infection can take place as a result of direct content with a carrier or indirectly through contact with the environment in which a carrier has been present, as the virus can survive from 4 to 72 h, depending on the type of surface [3]. SARS-CoV-2 is a β-coronavirus with a genome comprising a single strand of RNA. In its structure we can distinguish a nucleocapsid consisting of an N protein and RNA and a lipid bilayer envelope derived from the host cell, composed of both lipids and viral proteins (Figure 1).

In the case of SARS-CoV-2, the membrane receptor on the host cell is angiotensin-converting enzyme 2 (ACE2), which the virus uses as a gateway into cells. After the spike protein binds to ACE2, the virus enters the cell by endocytosis [5]. This is followed by cleavage of the S protein at two sites and penetration of the viral genome into the cytoplasm. When the virus penetrates a human cell, it initiates the production of specific viral polyproteins. Next, two viral proteases, i.e., papain-like protease and serine, cleave two polyproteins, ppla and pplab, leading to the formation of nonstructural proteins (NSPs 1–16). NSPs in turn build a replicase–transcriptase complex, which allows the virus to replicate its genetic material. This is followed by synthesis of mRNA, which is a matrix for the translation of the structural proteins S, E, M, and N. These proteins are relocated to the endoplasmic reticulum and then to the endoplasmic reticulum–Golgi intermediate compartment (E–GIC), where penetration of the viral RNA is followed by the formation of mature virions. Virions in the secretory vesicles penetrate the cell membrane, where their contents are released into the extracellular space, resulting in infection and an immune response [5]. Unfortunately, in some cases of COVID-19 the immune response becomes uncontrolled, and an excessive release of cytokines leads to a state of hyperinflammation, manifested as severe acute respiratory distress syndrome (SARDS) and then multi-organ failure [6]. Although the disease is accompanied by increased production of TNF-α, IL-1β, IL-8, IL-12, interferon-gamma inducible protein 10 (IP10), macrophage inflammatory protein 1A (MIP1A), and monocyte chemoattractant protein 11 (MCP1), a critical role is ascribed to another inflammatory cytokine—interleukin 6 (IL-6). IL-6 is produced by active leukocytes and exhibits activity within most cells and tissues [7]. A dramatic increase in the concentration of IL-6 in the body is manifested as high fever and multi-organ failure, which, when accompanied by conditions that already weaken of the body—e.g., chronic inflammation, multi-organ diseases, or mitochondriopathy—can even lead to the patient’s death [8].

### Angiotensin-Converting Enzyme 2 (ACE2)

Angiotensin-converting enzyme 2 is a protein present in many types of cells and tissues: in the lungs, heart, blood vessels, kidneys, pancreas, liver, and gastrointestinal tract. It is located in epithelial cells, which line tissues and form protective barriers. In the lungs, it is present in large quantities in type II pneumocytes [9]. ACE2 is a negative regulator of the renin-angiotensin system, facilitating transport of amino acids and the SARS-CoV and SARS-CoV-2 receptors [9]. ACE converts angiotensin I into angiotensin II, but also catalyzes the conversion of angiotensin II to angiotensin 1–7, with vasodilating, anti-inflammatory, antioxidant, and cytoprotective effects [10]. In this way ACE2 lowers blood pressure by catalyzing hydrolysis of angiotensin II to angiotensin 1–7. Hence, the ACE2 system is a very important protective pathway against heart failure occurring with a reduced ejection fraction, including against myocardial infarction and hypertension, and against pulmonary diseases and diabetes. Binding of ACE2 by SARS-Co-2 reduces the level of this enzyme in the lungs, which is a likely cause of the severe pulmonary symptoms observed in infected patients [9]. ACE2 also takes part in regulation of blood glucose levels [9]. Diet thus has a certain influence on the activity of angiotensin-converting enzyme; for example, a high-fat diet raises its level [11].

## 2. Risk Factors for Severe COVID-19

Most people infected with the SARS-CoV-2 experience no symptoms or only mild ones. However, there is a long list of factors that may cause a mild infection to transform into a life-threatening condition. The National Health Service (NHS) classifies risk factors for severe COVID-19 as high or moderate [12]. The high-risk group includes cancer patients, transplant recipients, and those with severe pulmonary disease. The other group of factors includes age (>70 years), obesity (BMI ≥ 40), and diabetes. Reports from many countries suggest that obese patients have poor prognoses with the disease. Obesity causes atelectasis and alveolar collapse due to increased pressure in the pleural cavity and reduces lung recruitment capacity [13,14]. According to the Centers for Disease Control and Prevention, the most common co-occurring diseases in COVID-19 cases are cardiovascular disease (32%) and diabetes (30%) [15]. Two thirds of patients in the United Kingdom with serious cases of COVID-19 were overweight or obese, and 99% of deaths in Italy were in patients with pre-existing diseases such as hypertension, diabetes, and heart disease [16]. These diseases are jointly referred to as metabolic syndrome (MetS). Metabolic syndrome encompasses a number of interrelated metabolic disorders, such as obesity, impaired glucose tolerance, insulin resistance and/or hyperinsulinemia, dyslipidemia, and hypertension. Their co-occurrence increases the risk of cardiovascular diseases caused by atherosclerosis and type 2 diabetes [17]. Doctors diagnose metabolic syndrome based on guidelines recommended by the International Diabetes Federation in 2009 [18].

One of the factors stimulating metabolic syndrome is insulin resistance, which is impaired sensitivity of tissues to insulin, the hormone regulating the glucose concentration in the blood. This occurs due to a defect of the insulin receptor, or much more often due to disturbances in processes signaling the binding of insulin to the insulin receptor [19]. Clinical observations that severe COVID-19 usually occurs in older patients, in men, and in patients with hypertension, elevated blood glucose levels, and abnormal results from liver function tests suggest that insulin resistance may play an important role in the course of COVID-19 [19]. Angiotensin-converting enzyme 2 (ACE2) is a potentially important molecular link between insulin resistance and severe COVID-19 [20,21]. It functions as a ligand through which coronaviruses bind to target cells [21]. ACE2 is expressed mainly in alveolar epithelial cells, pancreatic beta cells, and enterocytes of the small intestine [20]. As mentioned above, the primary physiological role of ACE2 is conversion of angiotensin 2, with vasoconstrictive and pro-inflammatory effects, to angiotensin (1–7), with a vasodilating effect [22]. Importantly, angiotensin 2 is the dominant component of the renin–angiotensin–aldosterone system (RAAS), which enhances insulin resistance and cardiovascular dysfunction. By degrading angiotensin 2, ACE2 protects against the effects of excessive activation of RAAS. The risk of insulin resistance is reduced owing to a decrease in cellular oxidative stress and enhancement of insulin signaling and glucose transport [23]. Expression of this enzyme is increased in people with diabetes. It is unclear, however, whether it is insulin or glucose itself that affects the ACE2 level. This distinction may be of clinical importance, because it could determine whether normalization of the blood glucose level or the insulin level should be prioritized to reduce ACE2 expression and thus the severity of COVID-19 [19]. It is likely that other mechanisms as well, independent of ACE2 expression, contribute to the severe course of COVID-19 associated with concomitant diabetes. In the pathophysiology of diabetes, particularly in patients with uncontrolled glycemia, the innate immune system and humoral resistance are impaired [24]. This means that the first line of defense against any infection, including by SARS-CoV-2, is ineffective. Diabetes also causes inflammation with an excessive cytokine response. People with diabetes infected with SARS-CoV-2 have been observed to have much higher levels of interleukin-6 (IL-6) and C-reactive protein (CRP) than individuals without diabetes [25]. Penetration of SARS-CoV-2 into host cells induces an inflammatory response, repeated recruitment of T-helper cells, and production of interferon gamma, which lead to a cytokine storm [26]. For this reason, given the cellular mechanisms induced by COVID-19 and the pathophysiology of diabetes, people with diabetes are more susceptible to the occurrence of a cytokine storm with potential organ damage [27]. Deregulated glucose concentrations in the blood also affect the course of respiratory diseases and inflammatory states. A study in COVID-19 patients with pre-existing type 2 diabetes showed that people with a regulated glucose level in the blood fared better than those with poor control of glucose levels. Having a normalized blood glucose concentration (glycemic variability from 3.9 to 10.0 mmol/L) was linked to fewer medical interventions, fewer cases of serious organ damage, and less mortality from all causes during hospitalization compared to patients with poorly controlled blood glucose concentrations (glycemic variability exceeding 10.0 mmol/L) [28]. Another study showed that hospitalized patients with hyperglycemia treated with an insulin infusion had a lower risk of death from COVID-19 than patients who did not receive an insulin infusion, most likely due to a decrease in inflammatory mediators [29].

It should be stressed here that in the case of diabetes, irrespective of the treatment applied, diet plays an enormous role. Regardless most diets, not only in our households but also in hospital wards and care homes, contain large amounts of simple carbohydrates. A systematic review comparing low-carbohydrate diets with low-fat diets showed that the low-carbohydrate diets were more effective at achieving control of glycemia, and at reducing both the short-term and the long-term risk of cardiovascular disease in patients with type 2 diabetes [30]. There is strong evidence that limiting carbohydrates in the diet is a safe and effective means of achieving control of glycemia and weight loss, and of reducing the need for medication to treat type 2 diabetes. In 2018, Diabetes Australia issued a statement claiming that there is reliable evidence that limiting consumption of carbohydrates can be a safe and effective meanss of reducing blood glucose levels, reducing body weight, and managing the risk factors of heart disease, such as an elevated cholesterol level and hypertension [31]. Furthermore, the American Diabetes Association in 2019 and Diabetes Canada in 2020 came out in support of low-carbohydrate diets as a real option for improving glycemia and potentially reducing medication doses for people with type 2 diabetes [32,33]. Limiting carbohydrates in the diet is a simple and safe intervention that quickly improves control of glycemia and can be implemented alongside the usual treatment in a medical or home environment. Although the pathophysiology of COVID-19 is multifactorial, insulin resistance is one of the strongest risk factors influencing impairment of metabolic functions. Nutritional counselling should be implemented in order to minimize the risk of complications from COVID-19 [31,34].

**Figure 2:**
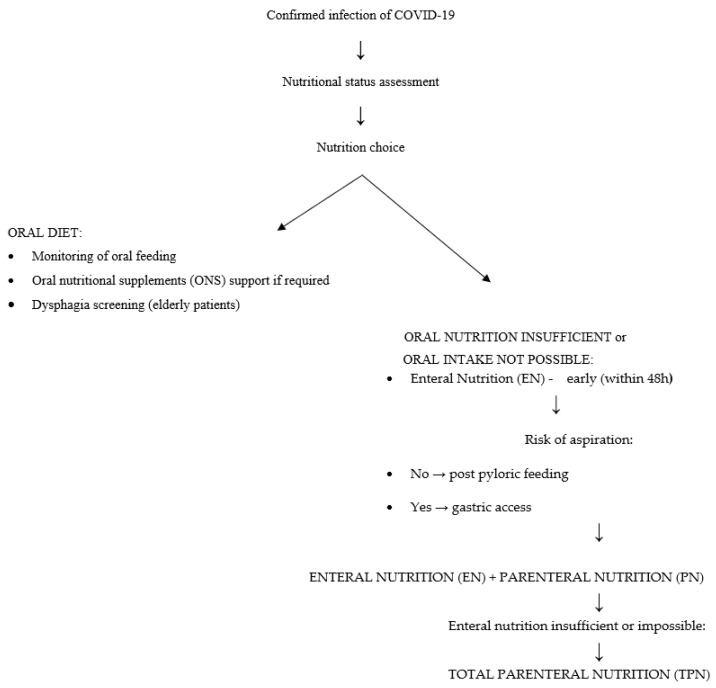
A diagram of dietary management in COVID-19 in hospital conditions. Modifications were based on the “ESPEN guideline on clinical nutrition in the intensive care unit [45]”.

A different problem is malnutrition, especially in older patients. It is both a short-term and a long-term prognostic factor of increased mortality. Nutrient deficiencies (overt or latent), generalized or pertaining to single nutrients, reduce the body’s immunity and thus can increase the risk of illness following infection with SARS-CoV-2. Nearly all hospitalized patients, including those with COVID-19, have poor nutritional status upon admission [34,35]. Malnutrition in patients is confirmed by low levels of lymphocytes, prealbumin, and albumin [35]. It is worth noting here that albumin itself does not depend exclusively on nutrition, but is part of several nutritional screening indicators [36]. A low albumin level has been linked to higher mortality rates, and a low prealbumin level may indicate a risk factor for rapid-onset respiratory failure and the need for mechanical ventilation [37,38]. COVID-19 patients often have elevated levels of acute phase proteins and CRP, which indicate ongoing inflammation in the body [39,40]. This may result in restriction of food intake, which is another factor leading to malnutrition. An inadequate diet, inflammation, and the resulting malnutrition impair the immune response in these patients, reducing their chances of fighting the infection and thus increasing mortality [41,42]. Moreover, patients with critical symptoms of COVID-19 are at higher risk of malnutrition due to the imbalance in energy intake and expenditure. Fever, mechanical ventilation, increased respiratory muscle work, and hypercatabolism increase the body’s energy consumption. A similar effect is exerted by metabolic disorders, such as impaired glucose metabolism—an increased blood glucose level, decreased oxidation, increased glycolysis and glycogenesis, and also decreased tissue sensitivity to insulin. This state can be exacerbated by inadequate intake of nutrients due to appetite loss, shortness of breath, and impaired consciousness. In addition, enteral nutrition and/or antiviral drugs may result in gastrointestinal symptoms such as nausea, diarrhea, and vomiting [40]. Many hospitalized patients have comorbidities that may be associated with swallowing disorders. In the course of pneumonia itself, problems with swallowing may occur in the form of dysphagia of the mouth and throat, which can significantly reduce appetite. Dysphagia in patients with pneumonia is correlated with higher mortality and a poorer prognosis [43]. Therefore, these patients require rapid nutritional support in hospital conditions. Nutritional screening should be performed for each patient admitted to hospital (in both mild and severe stages of disease) [40]. One useful tool is the NRS 2002 (nutritional risk screening) scale [44]. A result of ≥3 points indicates a nutritional risk requiring intervention. It is also important to repeat this type of examination regularly in order to monitor and control the nutritional status of patients and to assess the risk of dysphagia, especially in older patients. Where possible, nutrition by mouth is the best option [45]. The objective should be to maintain adequate intake of macronutrients and energy and meet the RDA for vitamins and minerals, because of their anti-inflammatory and antioxidant potential. If it is not possible to meet the requirements for protein and energy, patients should receive appropriately chosen oral dietary supplements [40,45,46]. However, these are not always well tolerated, and respiratory functions in patients may deteriorate during their use [40,45,46]. In that case, enteral or parenteral nutrition should be considered [40,45]. The choice of enteral feeding should be preceded by an assessment of choking risk. If the patient’s nutritional requirements cannot be met in this manner, parenteral nutrition should be considered [45]. The protocol of one Italian hospital involves a somewhat different procedure for early nutritional support of patients with COVID-19, in which parenteral feeding is implemented and enteral nutrition is deliberately omitted. This was explained by the fact that the presence of a nasogastric tube could negatively impact the effectiveness of treatment supporting respiratory function. The following is a simplified scheme according to the “ESPEN guideline on clinical nutrition in the intensive care unit” (Figure 2) [45,46].

## 3. Proposals for Specific Nutritional Requirements

In a patient whose calorie intake is reduced by illness, it is recommended to achieve a target supply of energy as quickly as possible. According to ESPEN (European Society for Clinical Nutrition and Metabolism) guidelines, nutritional support should be initiated within 24–48 h after admission to hospital, beginning with a small dose of energy for the first few days [45,47,48].

A meta-analysis carried out in patients in intensive care units (ICU) showed that early enteral nutrition within 24 h after admission to the ICU reduced mortality in patients compared to delayed nutrition [49]. In practice, resting energy expenditure (REE) can be calculated using a formula with the VCO2 measurement [47]. When it is not possible to directly measure VCO2, the energy requirement should be calculated according to body weight:

- for patients with normal body weight (BMI = 20–25 kg/m^2^) in critical condition, the recommended energy level is 25–30 kcal/day/kg BW.
- for overweight or obese patients (BMI = 25–30 kg/m^2^) in critical condition, the recommended energy level is 21 kcal/day/kg BW.
- for obese patients in critical condition, the recommended energy target is 11–14 kcal/day/kg BW for BMI 30–50 kg/m^2^ and 22–25 kcal/day/kg BW for BMI > 50 kg/m^2^ [47,50].

### Detailed Nutritional Strategies

Nutritional treatment can be based on a multistage scheme [45,50,51]:

- Patients whose condition allows for oral nutrition. The patient should receive a traditional oral diet with an energy value of 25–30 kcal/kg/day and protein content of 1.2–2 g/kg/day. The goal of the treatment is to meet 70% of the nutritional requirement within 3–7 days. Meals should be small, and if there is a choking risk a semi-liquid diet should be used. Nutritional education for the patient is recommended.
- Patients whose condition allows for oral nutrition, but with the risk that it will be inadequate. In addition to a traditional diet, the patient should receive oral nutritional supplements supplying 400–600 kcal. Increasing the amount of protein in the diet may reduce mortality. It is crucial to carefully monitor side effects, assess treatment effects, and dynamically adjust the treatment plan to the clinical course of COVID-19. Protein intake should be about 1.5–2.0 g/kg BW/day. When it does not meet the requirement, a standard protein preparation is recommended.
- Patients in critical condition, when oral nutrition is not possible and insertion of a nasogastric tube is required. Total enteral nutrition is implemented (10–20 mL/h; 10–20 kcal/h). The treatment goals are to prevent gastrointestinal complications during mechanical ventilation, stimulate intestinal immune function, and maintain intestinal barrier integrity and intestinal function.
- Patients in critical condition with a contraindication for enteral feeding, with no possibility of achieving an adequate energy level. Partial enteral nutrition and/or total parenteral nutrition should be implemented (<60% of energy; initially glucose in infusions of 5 mg/kg/min; fatty acids: infusion time > 8 h). An ‘all-in-one’ solution is recommended.

## 4. Food Components with Regard to Their Protective Effects in COVID-19 Patients

Dietary strategies aimed at preventing COVID-19 and dangerous pneumonia are continually being considered. Unfortunately, as in the case of drugs, studies in humans have not confirmed any specific food components to be effective in the case of COVID-19. We know that the immune system is the key to reducing the severity of COVID-19, and perhaps by modulating it in the right way we can save human lives with preventive measures. Numerous clinical studies have shown that nutraceuticals can beneficially stimulate the immune response in patients with various diseases, such as cancer or AIDS, and in healthy people at risk of viral infections [52,53,54,55]. Natural compounds are commonly recognized as valuable agents in the fight against viruses due to their structural diversity and safety. Many products consumed by people and used in traditional medicine have been shown to contain substances with anti-inflammatory, antibacterial, and antiviral properties, e.g., vitamin C in the fruit or juice of raspberries or elderberries, hesperidin in St. John’s wort, kaempferol and methylglyoxal in honey, allicin in garlic and onion, gingerols in ginger, curcumin in turmeric, and piperine in black pepper. However, there is no strong scientific evidence, nor are there any systematic literature reviews with meta-analyses indicating that herbs, spices, health-promoting food ingredients, or dietary supplements prevent infection with SARS-CoV-2, mitigate COVID-19 symptoms, or can even be used to treat infections, including severe “COVID pneumonia,” acute lung failure, a “cytokine storm,” clotting disorders, or multiple organ failure. Such connections can only be considered and hypothesized. Based on available in vitro evidence, greater understanding of the aggressiveness of COVID-19, and available data on other viruses, we present for consideration the following nutraceuticals and dietary strategies:

### 4.1. Elderberry

The elderberry (Sambucus nigra) may be useful against COVID-19 due to its capacity to stimulate the immune system and inhibit the replication of viruses, including human coronavirus NL63 (HCoV-NL63), which differs from COVID-19 but belongs to the same family of coronaviruses [56,57,58,59]. It could be especially helpful during the initial stage of coronavirus infection or for preventing infection. By inhibiting replication of the virus, elderberries significantly increase the production of pro-inflammatory cytokines (IL-1β, TNF-α, IL-6, and IL-8), especially TNF-α, which enhances the response of macrophages to a viral infection [60,61,62,63]. A review by the Natural Standard Research Collaboration concluded that there is level B evidence in support of the use of elderberries to treat the flu [61], which may or may not be of significance for the prevention of COVID-19. The typical dose of 2:1 elderberry extract is 10–60 mL a day for adults and 5–30 mL a day for children.

### 4.2. LC-PUFA

The concept of the “cytokine storm” refers to a reaction of the body manifested by an inability to defeat the virus, so that it begins to produce vast amounts of antibodies, which attack its own cells and tissues, instead of fighting only the pathogen. It has even been compared to drowning in the water used to extinguish a fire. Undurti N. Das [64] showed that unsaturated omega-3 and omega-6 acids may regulate inflammation, whereas deficiencies in unsaturated fatty acids, mainly EPA and DHA, may increase one’s susceptibility to viral diseases, including the novel coronavirus causing COVID-19. Omega-6 fatty acids are substrates for the production of pro-inflammatory prostaglandins and leukotrienes, which help the immune system to take control over invasion by pathogens [65]. The role of omega-3 fatty acids is to inhibit pro-inflammatory processes and suppress the immune response, e.g., by promoting the proliferation of Treg cells and inhibiting activation of neutrophils and monocytes [65]. In a pilot study [66], the risk of death from COVID-19 for subjects with the lowest concentrations of omega-3 fatty acids in the blood was four times as high as the risk for those with the highest concentrations. DHA and EPA exhibit strong anti-inflammatory activity, and both oral and intravenous intake of these acids may increase resistance to COVID-19 and improve the prognosis of infected patients. To reduce oxidative stress and the frequency of acute respiratory distress syndrome and sepsis, a mixture of fish fatty acids can be used: docosahexaenoic acid (DHA) + eicosapentaenoic acid (EPA) [48,50]. In the case of enteral feeding, 500 mg EPA + DHA/day is recommended, and in the case of parenteral nutrition, from 0.1 to 0.2 g per kg/day [48].

### 4.3. Selenium

Selenium plays an important role in protecting the respiratory system, especially against viral infections [67]. Deficiencies of selenium in the diet can affect the immune response and the pathogenicity of the virus [68,69]. Administration of selenium in combination with CoQ10 decreases the non-specific inflammatory response and mortality from cardiovascular causes [70,71]. When administered with acetylcysteine, selenium helps to achieve a normal level of intracellular GSH (reduced glutathione), which is responsible for the optimal level of GPX (glutathione peroxidase—one of the strongest antioxidant selenoenzymes). The GPX mimetic ebselen (a synthetic selenium compound) is a strong inhibitor of the main SARS-CoV-2 protease [72,73]. The role of selenium in normal immune system function is linked to its antioxidant properties and its ability to increase the production of interleukin IL-2, which exhibits immunoregulatory properties; i.e., it stimulates or inhibits immune reactions depending on current needs [74,75]. In addition, the beneficial effect of appropriate selenium doses on the immune system is important in patients infected with SARS-Cov-2, because it modulates secretion of IL-6, which plays a key role in the pathophysiology of the disease [74]. Selenium administration is recommended at doses from 100 to 200 µg a day [76].

### 4.4. Vitamin D

Vitamin D receptors are present in many immune cells and modulate the response to viral lung diseases. Vitamin D is thus a significant factor in protection against infectious respiratory diseases [77] and plays an important role in the prevention of COVID-19. Research has shown the infection rate to be higher in countries with higher latitudes and/or lower levels of vitamin D [78,79]. In another study, from Los Angeles, vitamin D deficiency was identified as a risk factor for positive COVID-19 tests [80]. The authors of a study from Cincinnati found links between vitamin D deficiency and hospitalization, disease severity, and death among patients in primary care and specialist clinics [81]. Severe vitamin D deficiency is often observed in patients in critical condition and seems to be linked to a poor prognosis [82]. It is associated with exacerbation of pneumonia, leading to acute respiratory distress syndrome with damage to the respiratory epithelium and hypoxia [82]. For prevention, long-term intake of vitamin D at dosages of ≤ 100 μg D3/day is recommended [83]. Vitamin D stimulates and modulates the immune response and protects the upper and lower respiratory tract from viral infection [84,85]. Cholecalciferol exerts an immunomodulatory effect by differentiating monocytes into macrophages and increasing their chemotactic capacity and phagocytic capacity [86]. Vitamin D deficiency has been shown to expose people to a high risk of viral respiratory infection [87]. Furthermore, vitamin D modulates T cell function; stimulates the production of anti-inflammatory IL-10; and reduces the production of pro-inflammatory IL-1β, IL-6, TNF-α, RANKL, COX-2, and nitric oxide [88]. Based on the latest scientific reports, oral supplementation with vitamin D may not only reduce the risk of SARS-CoV-2 infection, but also reduce mortality in patients with COVID-19 [89]. Recently published meta-analysis results indicate that the level of vitamin D does not affect the risk of infection, but that vitamin D deficiency is significantly linked to more severe disease and a higher risk of death [90]. Computer simulations were used to show that both forms of vitamin D (ergocalciferol and cholecalciferol), retinoids (vitamin A derivatives), and steroids “fit” the spike protein of the virus [91]. The SARS-CoV-2 spike protein has been shown to mediate entry into human cells by binding to the ACE2 receptor. People with high cholesterol levels be at greater risk of severe COVID-19 because cholesterol binds to the spike (S) protein in such a way that it exposes the ACE2-binding domain. Interestingly, vitamins A, D, and K, linolenic acid, and the drug dexamethasone bind to the S protein at another site, thereby stabilizing its closed conformation. This means that the region of the protein responsible for binding to the ACE2 receptor is not exposed, and the risk of infection decreases. In obese people, the vitamin D concentration in the blood is reduced (deposited in adipose tissue), and as mentioned above, the risk of severe COVID-19 is greater.

In our discussion of vitamin D, it should be borne in mind that the main source of all vitamins should be the diet. Among foods, the best source of vitamin D is fatty fish (on average 10 µg/100 g vs. 0.4 µg/100 g in the case of lean fish). Other products supplying vitamin D include eggs (on average 0.8 µg in one egg), margarine, and blended spreads—to which the addition of vitamin D is mandatory in Poland (maximum 7.5 µg/100 g). However, the effectiveness of the use of vitamin D for preventing and treating COVID-19 is still not fully known. The situation regarding scientific studies on this vitamin and coronavirus infection is highly dynamic, with new research papers appearing nearly every week. At the current stage of knowledge, although the apparent role of vitamin D in coronavirus infection has not been confirmed, it is believed that supplementation may have clinical benefits, but should only be implemented in controlled hospital conditions (for patients and medical personnel), to raise the concentration of vitamin D to exceed even 40 ng/mL. Some papers have concluded by recommending a daily intake of 2000–4000 IU of vitamin D to reach a concentration of 30 ng/mL to prevent infection, and stating that infected patients should be given a bolus dose (50,000–200,000 IU), after which the dose should be adjusted to maintain a concentration of 40–60 ng/mL of the vitamin in the blood [92,93,94,95,96]. Further studies, especially randomized trials, may provide specific practical solutions.

### 4.5. Vegetables and Fruit

According to well-known recommendations, half of one’s daily diet should consist of vegetables and fruit. We should consume at least five portions of fruit and vegetables every day, predominantly vegetables (3–4 portions of vegetables and 1–2 portions of fruit). Ideally, they should be of different colors and raw or minimally processed. This may be linked to a reduced risk of infection with SARS-CoV-2 due to the presence of antiviral and immunostimulatory compounds in these foods, particularly flavonoids. Many flavonoids have been shown to reduce signaling of the NLRP3 inflammasome in vitro, thereby reducing NFkB expression and indirectly expression of TNF-α, IL-6, IL-1β, and IL-18 [97]. Inflammasomes are a group of intracellular complexes contained in the cytosol and constituting an element of nonspecific immunity. They are responsible for detecting damage-associated molecular patterns (DAMP) and the presence of pathogens (pathogen-associated molecular patterns (PAMP)), and for the production of pro-inflammatory cytokines [98]. The mechanism of NF-kB activation has several steps. Normally, NF-kB is inactive and blocked by proteins inhibiting IkB. The virus activates NF-kB, causing degradation of IkB by IkB kinase (IKK); NF-kB enters the nucleus and induces DNA transcription. Once NF-kB has moved into the cell nucleus, the coronavirus causes it to bind to DNA and activate genes of the inflammatory response, causing a “cytokine storm”—i.e., overproduction of inflammatory cytokines (IL-1, IL-6, and TNF-α), molecules (ICAM), and enzymes (COX-2)—which in turn leads to mass migration, infiltration, and accumulation of immune cells in lung tissues. This is followed by severe cellular hypoxia, which causes lung damage. Therefore, inhibition of NF-kB may be beneficial for controlling inflammatory states. Attempts can be made to block NF-kB naturally, using flavonoids such as the following.

Epigallocatechin gallate and its derivatives epigallocatechin, epicatechin gallate, and epicatechin, found in green tea, exhibit antiviral and anti-cancer properties. They most likely inhibit the early stages of infection, i.e., adhesion, penetration, and membrane fusion, by interfering with viral membrane proteins. To enhance the virucidal effect, i.e., the binding affinity of epigallocatechin gallate to viral particles, it is recommended to use an epigallocatechin gallate derivative and a fatty acid. The fatty acid on the phenolic hydroxyl group should increase the permeability of viruses and cell membranes [99,100,101].

Quercetin is present in ginkgo biloba, and in fruits and vegetables such as apples, berries, grapes, onions, shallots, and tomatoes [102,103]. In a clinical trial with patients with chronic systemic inflammation (CSI) in stable coronary artery disease (CAD), quercetin exhibited anti-inflammatory activity with decreased CSI indicators. Quercetin decreased gene expression and serum levels of IL-1β, IL-6, IL-8, and TNF-α, and the transcriptional activity of NF-kB in mononuclear blood cells. The doses in the trial ranged from 100 to 1000 mg/day, but due to poor bioavailability (2–3%), doses of 500–1000 mg/day are recommended. It should be stressed that both quercetin and epigallocatechin gallate act as antioxidants and signaling molecules, and the functions of many enzymes activated by polyphenolic compounds depend on zinc. Flavonoids have also been shown to act as zinc ionophores, transporting zinc cations through the plasma membrane [104]. In theory, this could enhance the antiviral effects of zinc.

Baicalin and wogonoside are found in Chinese skullcap (Scutellaria baicalensis Georgi). Several studies have found very promising antiviral properties, including NF-kB inhibition. Moreover, baicalin exhibits virucidal activity against extracellular viral particles, which is important in minimizing the risk of severe disease [105,106].

Liquiritigenin, present in liquorice Glycyrrhiza glabra, inhibits the inflammatory response induced by an elevated glucose level, thereby reducing the expression and secretion of IL -6 and IL-1β in the mesangial cells of the renal glomeruli (HBZY-1). Likirytygenin has also been shown to inhibit activation of NF-κB inflammasome pathways induced by excess glucose [107]. Compounds contained in liquorice root can thus potentially inhibit viruses such as HIV-1, HCV, HSV, EBV, and coronaviruses.

Myricetin, which is found in tomatoes, oranges, nuts, and berries, inhibits the formation of the NLRP3 inflammasome, which plays a key role in the innate immune response and the pathogenesis of many inflammatory diseases. An in vivo study using murine models of sepsis induced by lipopolysaccharides (LPS) and alum-induced peritonitis showed that myricetin inhibits the formation of the NLRP3 inflammasome [108]. Moreover, due to the pleiotropic properties of myricetin (anti-inflammatory, antioxidant, autophagous, and reduction of the formation of amyloid plaques and tau protein phosphorylation), myricetin is a promising compound for the treatment of neurodegeneration of the brain caused by ischaemia and full-blown dementia [109].

Curcumin, found in Curcuma longa, is not well absorbed. Even high doses do not cause a significant increase in the serum level. However, administration together with piperine and fat increases its intestinal absorption. This substance has anti-inflammatory effects, suppressing cytokines and reducing expression of the enzymes COX-2 and iNOS. Curcumin also suppresses the production of pro-inflammatory cytokines TNF-α, IL-1, IL-2, IL-6, IL-8, and IL-12 [110,111]. However, its best described anti-inflammatory mechanism of action is inhibition of NF-kB signaling. Curcumin has been shown to inhibit the degradation of IkB and subsequently the translocation of the protein p65 NF-kB to the nucleus. Curcumin also blocks the signal leading to activation of IKK [112].

Resveratrol (found in black, red, and green grapes, strawberries, raspberries, bilberries, cranberries, and currants) exhibits anti-inflammatory activity. In vitro studies have shown that resveratrol more effectively inhibits NF-kB than glucocorticosteroids or dexamethasone [113,114]. The anti-inflammatory properties of resveratrol have been shown to be strong enough to reduce expression of the enzyme COX-2, even following administration of a pro-inflammatory agent [114]. This effect is a reflection of the ability of resveratrol to inhibit NF-kB and thereby reduce pro-inflammatory gene expression [115,116]. Due to its ability to modulate platelet activation and aggregation, and factors associated with the coagulation cascade, resveratrol seems to be an attractive pharmacotherapeutic agent for the fight against COVID-19. It could be an auxiliary treatment and alleviate symptoms of thrombosis and systemic inflammation [116]. Elevated levels of circulating inflammatory cytokines are often associated with abnormal clotting parameters in patients with COVID-19. Although procedures with low-molecular-weight heparin (LMWH) have reduced mortality in patients with severe COVID-19, additional therapeutic strategies are needed. A possible alternative is the use of natural compounds to prevent and reduce risk factors associated with pre-existing conditions and comorbidities. Resveratrol, owing to its anticoagulant and anti-inflammatory properties, can be a promising adjuvant for the prevention and treatment of COVID-19.

### 4.6. Vitamin C

Vitamin C takes part in physiological processes associated with immunity and in the blood clotting process, which is of importance in the course of COVID-19. In order for the body to defend itself against viruses, it must have an adequate level of vitamin C. Like flavonoids, ascorbic acid inhibits activation of the NLRP3 inflammasome [103]. Clinical trials have shown that vitamin C reduces the frequency, duration, and severity of the common cold and the incidence of pneumonia. Typical daily doses of vitamin C range from 500 to 3000 mg, and even higher doses are used in periods of acute infection. It is theoretically possible that vitamin C affects COVID-19 [117]. In a clinical trial, during infection with the Epstein–Barr virus (EBV), reduced levels of anti-EBV IgG and IgM antibodies were noted during intravenous vitamin C treatment [118]. In the case of acute respiratory distress syndrome (ARDS) induced by rhinoviruses/enteroviruses in 2017, high-dose intravenous vitamin C (HDIVC) was associated with rapid resolution of lung damage [119]. Many studies have confirmed the beneficial effects of high doses of vitamin C to treat lung damage, alleviate severe sepsis, and reduce the durations of stays in intensive care units and of mechanical ventilation in ARDS patients [120,121,122,123,124]. Other meta-analyses, however, have not found that high-dose intravenous vitamin C (6 g/day) improves the condition of patients with COVID-19 [125].

### 4.7. Melatonin

The use of melatonin can significantly reduce the levels of pro-inflammatory cytokines (TNF-α, IL-1β, IL-6, and IL-8) and increase the levels of anti-inflammatory cytokines, thereby stabilizing the work of the immune system [126]. Melatonin improves the proliferation and maturation of T and B lymphocytes, granulocytes, and monocytes. Melatonin produced in the lungs can be helpful for fighting SARS-CoV-2. It not only has strong preventive properties, but also inhibits the spread of the coronavirus in the body, according to the findings of researchers from the University of São Paulo (USP) [127]. Experts suggest that it could be used to produce a drug to treat COVID-19. Melatonin has been shown to inhibit NFkB activation and help to inhibit NLRP3 inflammasomes, which are linked to lung disease induced by infections. COVID-19 activates NLRP3s and enhances their effect, whereas melatonin reduces their expression levels and significantly decreases the penetration of macrophages and neutrophils into the lungs [128]. The reduction in melatonin production with age is one of the mechanisms explaining why children do not have severe symptoms as often as older people. Shorter sleep increases the risk of infection for many diseases. A lack of sleep, which is particularly important in the case of COVID-19, increases levels of the monokine CXCL9 induced by interferon, which increases lymphocyte infiltration and is involved in activation of the NLRP3 inflammasome [129]. Melatonin also reduces oxidative lung damage and recruitment of inflammatory cells during viral infections [126]. Typical doses of melatonin vary widely from 0.3 to 20 mg (this last dose is applied in cancer treatments).

### 4.8. Zinc

Zinc takes part in regulating the inflammatory response by influencing the activity of leukocytes and lymphocytes, including their proliferation, differentiation, and maturation, and also has direct antiviral effects [130]. Coronaviruses seem to be susceptible to the antiviral activity of zinc. Zinc can prevent the coronavirus from entering cells and seems to reduce its virulence [131,132]. A deficiency of zinc causes dysfunctional humoral and cellular immunity [133]. In older people, a low Zn level (serum level < 0.7 mg/l) has been shown to be a risk factor for pneumonia [134]. A long-term zinc deficiency exacerbates inflammation and biomarkers of the inflammatory state [135]. Combined administration of zinc and pyrithione, even in small concentrations, inhibited replication of the SARS coronavirus (SARS-CoV) in vitro by inhibiting the activity of RNA polymerase [136]. Consequently, zinc supplementation may influence not only the overactive inflammatory response associated with COVID-19, but possibly also SARS-CoV-2 itself [137]. In the long term, intake of ≤25 mg/day is recommended, because high zinc intake can disturb the balance of copper. The typical daily dose of zinc is 15–30 mg from lozenges, which directly protect the upper respiratory tract. Testing to determine the level of zinc prior to supplementation is essential [76].

### 4.9. The Microbiome—Probiotics

Given that SARS-CoV-2 has receptors in the intestinal endothelium, infection will reduce the expression of ACE2 in the gastrointestinal tract, and thus by various mechanisms may lead to intestinal dysbiosis [138]. The number of ACE-2 receptors in the duodenum increases with age, which suggests a potential entry mechanism for SARS-CoV-2 [139] and effects on its infectiousness and the severity of COVID-19 [140,141]. Research has detected the SARS-CoV-2 virus in anal swabs and stool samples in nearly 50% of patients with COVID-19, which suggests that the digestive tract may be an extrapulmonary site of replication and activity of the virus [6,142]. Moreover, an elevated level of calprotectin has been noted in the stool of COVID-19 patients with diarrhea, which is an indicator of the inflammatory response in the intestines [143]. During COVID-19 in patients in a severe/critical state of health, levels of pro-inflammatory cytokines IL-6, IL-10, IFN, and TNF-α are elevated [144]. Due to intestinal dysbiosis during COVID-19, the populations of commensal bacteria have been shown to be replaced by pathogenic species. For example, higher levels of **Klebsiella, Streptococcus, and Ruminococcus gnavus** in COVID-19 patients have been correlated with increases in the levels of pro-inflammatory cytokines (IFN-γ and TNF-α), leading to a cytokine storm and activation of T helper cells (Th1) [145]. Many opportunistic pathogens have been detected in stool samples from COVID-19 patients, including Streptococcus, Rothia, Veillonella, Erysipelato clostridium, Actinomyces, Enterococcus faecalis, Escherichia coli, and Klebsiella pneumoniae; and pro-inflammatory bacteria, such as Coprobacillus, Clostridium ramosum, Collinsella aerofaciens, Collinsella tanakaei, Streptococcus infantis, and Morganella morganii [140,146,147]. An altered composition of intestinal bacteria due to an intestinal infection can thus play a role in regulating our immune response during COVID-19, especially given that increased presence of Actinomyces viscosus of pulmonary origin has also been detected in the intestines [140]. The effect of the intestines on the immune system and the gut–brain axis itself can strongly influence the speed of recovery from COVID-19 [148]. Unfortunately, COVID-19 in the intestines can also have long-term health consequences, especially since the loss of useful microbes persists in most patients despite the removal of SARS-CoV-2, which suggests that SARS-CoV-2 infection and/or hospitalization may involve a long-term effect on the microbiome [140]. There is a need for further studies of the effects of SARS-CoV-2 on the intestinal microbiome during and after COVID-19. In particular, it is worth asking whether the use of probiotics would have health benefits. Probiotics are known to help to prevent gastrointestinal diseases, diarrhea following antibiotic treatment, sepsis, and respiratory tract infection (RTI) [149]. A randomized trial has also shown that in mechanically ventilated patients there are far fewer cases of pneumonia following administration of probiotics (Lactobacillus rhamnosus GG or live Bacillus subtilis) compared to controls [150]. It can be presumed that administration of probiotics would cause changes in the balance between Th1 and Th2 cells, and thus reduce the cytokine storm and severity of COVID-19 [151]. However, there is a concern about the possibility of opportunistic pathogen infections, especially in people with comorbidities such as cancer, leaky gut syndrome, or diabetes, and in organ transplant recipients [152]. Some probiotic strains take advantage of weakened immunity to transform into opportunistic pathogens, causing acute pneumonia, endocarditis, or sepsis [141]. There is a need for studies encompassing the effects of both diet and probiotics on the severity of COVID-19.

## 5. General Principles—Nutritional Prophylaxis

Obesity and the chronic diseases associated with it (diabetes, metabolic syndrome, and cardiovascular disease) can predispose patients to a more severe course of COVID-19 [153,154,155]. This is linked to chronic inflammation and the associated production of mediators, immune dysregulation, oxidative stress, endothelial dysfunction, and equally importantly—increased expression of the above-mentioned ACE2 [154,155,156]. There are also data indicating that adipose tissue is a storage site for viruses. All of these factors can contribute to poorer COVID-19 treatment results and reduce survival rates [157]. The adoption of good nutritional habits and a healthy, balanced diet are well known to be extremely important for preventing and treating obesity. Unfortunately, the COVID-19 pandemic has led to a profound change in eating behavior, even among inhabitants of Mediterranean countries, whose diets are regarded as models of good nutrition. Isolation at home has often been associated with the accumulation of non-perishable, highly processed food products and more difficult access to fresh food. In addition, many people have stopped exercising and begun to lead sedentary lifestyles [31]. The optimal nutritional model is still considered to be the Mediterranean diet or the similar DASH diet [158,159,160]. They can prevent obesity and are thus effective in preventing a more critical course of COVID-19. These diets are based on fresh, unprocessed plant-based foods, such as vegetables (including the seeds of legumes), fruit, and wholemeal products [158]. The plant products (fruit, vegetables, nuts, and olive oil) constituting the basis of the Mediterranean diet are a significant source of bioactive polyphenolic compounds. These compounds, especially flavonoids and their metabolites, exhibit pleiotropic health-promoting effects, especially in cardiovascular and metabolic disorders [159,160]. Owing to their antioxidant, anti-inflammatory, and anticoagulant properties, they are becoming even more important in the face of the severe inflammatory and prothrombotic state associated with COVID-19 [161,162]. They mitigate the immune response, increase antioxidant defense, improve vascular reactivity, and reduce tissue inflammation and cell infiltration. They appear to exert these beneficial effects by preventing activation of the NF-κB and NADPH signaling pathways and by lowering the levels of IL-6 and TNF-α [163,164]. In addition, the ellagic acid contained in some fruits and nuts exerts effects through interaction with the microbiota and epigenetic regulation [159]. Therefore, attempts to exploit the protective effects of flavonoids or other phytochemicals in the treatment of COVID-19 are extremely important [163]. In nutritional “anti-covid” prophylaxis, it is recommended to limit consumption of meat and animal fats, in order to reduce intake of saturated fats conducive to the development of low-grade inflammation [165]. Foods that should be incorporated in the diet include reduced-fat dairy products, vegetable fats (except for tropical fats—coconut and palm oil), and fish fat, which contains unsaturated fatty acids with proven immunomodulatory effects [166]. N-3 polyunsaturated fatty acids in seafood (shellfish, algae, and fish) and flax seeds support the immune system by activating both specific and nonspecific immune mechanisms [167]. N-9 monounsaturated fatty acids, present in olive oil and nuts, have antioxidant, antibacterial, and antiviral effects [168]. Experts also suggest avoiding salt and replacing it with herbs and spices. It is also recommended to exclude excessive amounts of sugar, ubiquitous in sweetened beverages, sweets, and highly processed foods. In the context of COVID-19, the roles of minerals such as zinc and vitamins C, D, and A are emphasized, as is adequate hydration of the body [158]. In addition, in considering the effect of diet on the body in a state of illness, we must not forget our mental health needs. Constant stress disrupts immune regulation and is associated with increased levels of pro-inflammatory cytokines, such as IL-6 [169]. Severe stress in mice has been shown to increase IL-1β by activating the NLRP3 inflammasome [170]. Various techniques, such as meditation, breathing exercises, and visualization, can be helpful for reducing stress and the levels of inflammatory cytokines.

## 6. Conclusions

Advanced age and comorbidities such as cardiovascular disease, diabetes, chronic respiratory diseases, obesity, hypertension, and cancer are linked to higher mortality from pneumonia induced by COVID-19. A healthy, well-balanced diet has a beneficial effect on these conditions that predispose one to COVID-19 and its consequences. A diet with high antioxidant, anti-inflammatory, and immunomodulatory potential may constitute prophylaxis mitigating the severity of COVID-19. For this reason, there is a need for more studies to determine the potential of specific diet components to prevent COVID-19 and improve parameters of infection during illness. In the case of COVID-19 patients that are hospitalized, in critical condition, and in intensive care units, it is recommended to assess their nutritional status and implement rapid and appropriate nutritional care. Nutritional support should include adequate intake of all nutrients—especially proteins, n-3 fatty acids, microelements (selenium and zinc), and vitamins, especially D and C—and a caloric value adjusted to the patient’s needs.
